# Ultraviolet Radiation-Induced Skin Aging: The Role of DNA Damage and Oxidative Stress in Epidermal Stem Cell Damage Mediated Skin Aging

**DOI:** 10.1155/2016/7370642

**Published:** 2016-04-11

**Authors:** Uraiwan Panich, Gunya Sittithumcharee, Natwarath Rathviboon, Siwanon Jirawatnotai

**Affiliations:** Laboratory for Systems Pharmacology, Department of Pharmacology, Faculty of Medicine Siriraj Hospital, Mahidol University, Bangkok 10700, Thailand

## Abstract

Skin is the largest human organ. Skin continually reconstructs itself to ensure its viability, integrity, and ability to provide protection for the body. Some areas of skin are continuously exposed to a variety of environmental stressors that can inflict direct and indirect damage to skin cell DNA. Skin homeostasis is maintained by mesenchymal stem cells in inner layer dermis and epidermal stem cells (ESCs) in the outer layer epidermis. Reduction of skin stem cell number and function has been linked to impaired skin homeostasis (e.g., skin premature aging and skin cancers). Skin stem cells, with self-renewal capability and multipotency, are frequently affected by environment. Ultraviolet radiation (UVR), a major cause of stem cell DNA damage, can contribute to depletion of stem cells (ESCs and mesenchymal stem cells) and damage of stem cell niche, eventually leading to photoinduced skin aging. In this review, we discuss the role of UV-induced DNA damage and oxidative stress in the skin stem cell aging in order to gain insights into the pathogenesis and develop a way to reduce photoaging of skin cells.

## 1. Introduction

Skin serves as the major protective organ of the body. This protection can be compromised by aging of the skin, a condition normally associated with skin inflammation, impaired wound repair, and increased risk of skin cancers [1, 2]. Skin aging is defined as a continuous loss of certain characteristics present in juvenile skin, including decreased skin elasticity and pigmentation, and loss of ESCs [3–5]. Skin aging is a multifactorial process that involves genetic and environmental factors. A variety of environmental stresses, particularly UV light, can damage sun-exposed areas of the skin, such as the face and neck, and accelerate premature aging [6]. Skin aging that is associated with UVR exposure is referred to as photoaging.

Adult tissues, including skin epidermis, gastrointestinal epithelium, and the hematopoietic system, have a high rate of cell turnover. To maintain their functions and integrity, the physiological process of maintaining tissue homeostasis is attributed to a constant number of cells in renewing organs. ESCs are essential for the maintenance and regeneration of skin tissues [7].

Adult skin is composed of a diverse organized array of cells emanating from different embryonic origins. During development, skin is derived from embryonic origins of cell types from different germ layers. Epidermis and dermis are developed from ectoderm and mesoderm, respectively. The epidermis develops from embryonic surface ectoderm, which starts as a single layer of unspecified progenitor cells covering the embryo after neurulation and becomes the epidermal basal layer [8]. The epidermal basal layer is enriched with ESCs. Thus, cells in this layer give rise to all epidermal structures, including a stratified epidermis (also called interfollicular epidermis) and epidermal appendages, such as hair follicles, sebaceous glands, and sweat glands. The underlying dermis is derived primarily from mesoderm under the ectoderm. The mesoderm is the major source of mesenchymal stem cells that give rise to collagen-producing fibroblasts (a component of blood vessels that provide nutrients to skin), subcutaneous adipocytes, and immune cells in the skin.

Dermal fibroblasts are the main mesenchymal cell type in dermis. Substructurally, they were shown to be derived from the upper dermis and lower dermis. The fibroblasts from the former contribute to hair follicle formation, while the fibroblasts from the later produce fibril extracellular matrix (ECM). ECM from dermal fibroblasts plays a crucial role in structural integrity and repair of the skin and wound healing [9].

Skin is also populated by specialized cells, including melanocytes and sensory nerve endings of the skin that are derived from neural crest cells. Overall, approximately 20 different cell types reside within the skin [8, 10].

ESCs are defined by their ability to self-renew and differentiate into different cell lineages belonging to the skin [11]. ESCs are capable of differentiating into the entire set of cells that comprise the skin. Thus, the epidermis is used for skin graft to replace damaged or missing skin [12]. ESCs were shown to be able to develop into three distinct layers of epidermis: spinous layer, granular layer, and cornified layer (or stratum corneum, composed of dead, flattened, and anucleated cells). ESCs were also shown to be capable of differentiating into multiple skin cell lineages, including mature and specialized keratinocytes, sebocytes, or pigmented melanocytes [13, 14].

In addition to the interfollicular stem cell, ESCs include stem cells in hair follicles, the hair follicle stem cells (HFSCs) that reconstitute hair follicles, and play role in wound healing [15]. Another type of stem cells in the epidermis is melanocyte stem cells (MSCs), which are intermingled with HFSCs in the hair bulge. MSCs generate mature melanocytes that produce melanin, which absorbs ultraviolet (UV) light to prevent DNA damage and gives skin and hair their distinctive colors [16].

Microenvironment within skin is important for maintenance of stem cells. The microenvironment provided from the complex structure of ECM and basement membrane form integral stem cell niches for the ESCs [17]. Adult ESCs residing within the niches remain there for self-renewal, whereas their progeny, committed to differentiation, leave the basal call layer and migrate towards the epidermal surface [13]. This made it relatively useful to track each lineage along the steps of differentiation. Thus, skin has served as one of the ideal models for studying stem cell differentiation and the interaction between stem cells and their respective microenvironments [18].

Because of their exposure to DNA damaging conditions, skin stem cells are innately highly resistant to the aging process and DNA damage and carry highly active DNA repair mechanisms [19–21]. Nevertheless, ESCs can be impaired in advanced age [22] and are vulnerable to repetitive exposure to UVR [23–25]. If not repaired properly, damaged DNA may result in mutation or chromosomal rearrangements, which negatively influences self-renewal and potency of stem cells and promotes skin aging and/or cancer formation [26].

To prevent photoaging and skin cancers, reduced exposure to DNA damaging agent (UVR) and well-controlled DNA repair mechanisms in ESCs are required to prevent photoaging and DNA damage-associated skin diseases, such as cutaneous basal cell and squamous cell carcinoma (BCCs and SCCs) [27].

While the role of UVR-induced DNA damage and oxidative stress in the skin aging involving ESC changes will be the focus of this review, it should also be noted that other factors may also contribute to this process. Skin in the area not normally exposed to UVR also can exhibit aging phenotype. Factors such as chronological shortening of skin cell telomere [28], increased inflammatory responses, and accumulation of senescent cells could contribute to skin aging by impairing stem cell function and homeostasis [29].

## 2. UV-Induced DNA Damage in Skin Cells

Repetitive exposure to solar UVR is among the principal environmental factors that can hasten the aging process of the skin, accompanied by progressive impairment of epidermal stem cell function [30]. UVR is a natural component of sunlight and is invisible to human eye. Three types of UV are categorized according to their wavelength: UV-A (315–400 nm), UV-B (280–315 nm), and UV-C (100–280 nm). UV-C is completely filtered out by the ozone layer of the Earth's stratosphere. Only UV-A and UV-B can reach the earth surface. However, climate change and depletion of the ozone layer may lead to increases in UV radiation at ground-level. UVR is known to be a mutagen; long-term overexposure to sunlight is associated with photoaging and formation of skin cancers [31]. Interestingly, both photoaging and cancer-inducing effects of UVR are mediated through UVR's direct and indirect toxicity to the DNA [32].

The direct effect of UV irradiation occurs when DNA absorbs photons from UV-B. This results in structural rearrangement of nucleotides that then leads to defects in the DNA strand. Cyclobutane pyrimidine dimers (CPD) and pyrimidine (6-4) pyrimidone (6-4 photoproducts, 6-4 PPs) are the major products of UV-B-induced DNA damage [32]. Direct absorption of UV-B photons induces cycloaddition between C5-C6 of two adjacent pyrimidine bases and changes them into CPD. Meanwhile, covalent bond formation of C6-C4 of two adjacent pyrimidine bases generates 6-4 PPs photoproduct, which further converts to its Dewar valence isomer upon UV excitation at 314 nm. The hotspot within these UV-induced DNA lesions is where tandem pyrimidine residues form (TT, TC, CT, and CC) [33]. At this point, cells will respond by promptly halting cell division to prevent further DNA damage and allow the DNA repair mechanism to start. In lower species, photolyase enzyme is employed to repair DNA by removal of UV-induced DNA lesions in a specific manner. The enzyme binds CPD or 6-4 PPs at lesion sites and splits the dimers back to undamaged bases. However, human and other mammalian cells no longer possess this enzyme. The main UV-induced DNA damage response in humans is the nucleotide excision repair (NER) pathway. There are 9 major proteins that function as NER in mammalian cells. RAD23s, RPA, ERCC1 proteins, and others also participate in nucleotide excision repair [34, 35]. Deficiencies of these proteins lead to diseases associated with DNA damage and premature skin aging [36].

NER starts when XPC forms complex with CETN2 and RAD23 at distorted DNA sites and DNA is unwound by XPB. The damaged DNA is then stabilized by XPA, XPB, XPD, and RPA (single-stranded DNA binding proteins). RPA will then activate structure-specific endonucleases that contain XPG and ERCC1-XPF, followed by base excisions. Gaps are filled by DNA polymerase and fragments are sealed by DNA ligase. In addition to DNA repair pathway defects, mutations are also caused by error-prone DNA polymerase, which tends to add a residue at the position where the specific base is missing, thus creating “UV signature” mutations of C→T and CC→TT transition, as a result of UV irradiation [37].

UV radiation also indirectly damages DNA. Absorption of UV-A photons drives electrons and energy transfer from cellular photosensitizers, such as porphyrins, bilirubin, melanin, and pterins, to oxygen molecules creating the radical singlet oxygen (^1^O_2_) anion [32]. Consequently, the singlet oxygen anion induces guanine moiety oxidation followed by structural rearrangement and 8-oxo-7,8-dihydroguanine (8-oxo-G) and 8-oxo-7,8-dihydro-2′-deoxyguanosine (8-oxo-dG) formation, which is the biochemical marker of UV-A-induced DNA damage. 8-oxo-G and 8-oxo-dG tend to pair with adenine instead of cytosine leading to C-T transition mutation [38, 39]. Protective mechanisms against UV-induced oxidative damage acquire both antioxidative pathway and base excision repair (BER) pathway. Removal of 8-oxo-dG by BER is performed by a key enzyme, human 8-oxoguanine DNA glycosylase (hOGG1), which specifically recognizes and cleaves glycosidic bond from the DNA strand creating abasic (AP) sites. Subsequently, missing nucleotides are restored by DNA polymerase and gaps are sealed by DNA ligase. Accordingly, depletion of hOGG1 by microRNA inhibits the repair of UV-A-induced 8-oxo-dG in keratinocyte HaCaT cells [40].

## 3. UV-Induced Depletion of Skin Stem Cells: A Multipronged Attack on Skin Stem Cells

Several studies have investigated the role of DNA damage on function and genetic instability of ESCs [27, 41, 42]. Human ESCs are sensitive to DNA damaged lesions. Ionizing radiation-mediated DNA damage reduced colony-forming potential and viability of skin stem cells and destabilized the stem cell microenvironment in the bulge region of hair follicles. As a consequence, MSCs aging with related hair graying was observed [43]. It was shown that damage to ESC DNA by low-dose ionizing radiation significantly induced DSBs-mediated chromatin modifications and that DSBs may also be passed on to their differentiated cell lineages [44]. Persistent exposure to UVR also increased DNA damaged lesions and mutations and led to premature aging or carcinogenesis of the skin [45].

Links between UVR exposure and skin aging can be observed when the DNA repair mechanism becomes dysfunctional. Quite often, defects in DNA repair systems of stem cells accelerate the accumulation of genome instability in lineage-primed progenitor cells during aging [46]. As described previously, NER is one of the most versatile DNA repair systems for eliminating a variety of helix-distorting DNA lesions induced by UVR [47]. Aberrance in this pathway contributes to UV hypersensitivity.

Mouse models genetically engineered to contain null allele of XPC shared skin aging phenotypes [48]. Genetic correction of keratinocyte SCs from NER-deficient XPC patients was observed to restore DNA repair capacity and cell survival following UVR, as well as providing long-term growth potential of stem cells [49]. Studies of human genetic disorders collectively known as progeroid syndromes (PS) (characterized by conditions that resemble premature aging) found mutations in the germ cell line or stem cells of genes in DNA repair pathways. PS-associated premature aging affects all tissues, including the skin, causing skin atrophy, hair graying, and abnormal pigmentation [36]. Patients with PS also develop other systemic diseases such as atherosclerosis, osteoporosis, and diabetes mellitus at an early age [50]. Some DNA repair defects implicated in PS are found in Werner's syndrome (WS), Hutchinson-Gilford progeria syndrome (HGPS), dyskeratosis congenita, xeroderma pigmentosum (XP), and Cockayne syndrome (CS) [51, 52]. All of these rare syndromes associate with depletion and dysfunction of adult stem cells and their progenies in various tissues, including the skin. The most well-studied inherited disorder, among these, is XP, involving the mutation of XPs genes and defective NER. XP patients, whose genome harbors XPs gene mutations, are generally hypersensitive to solar UVR, accumulate increased level of mutations, and develop skin aging and skin carcinoma by an early age with an approximate 1,000-fold increased risk [53, 54].

In addition, evidence of NER role in skin aging is observed in physiological aging. Decline in NER function associated with increasing age is speculated to contribute to the onset of aging manifested in various tissues including the skin.

Telomeres are nucleoprotein complexes that cap and save the ends of chromosomes from degradation and abnormal recombination. Telomeres shorten with each cell division and progressive telomere shortening ultimately results in cellular senescence. It is thus suggested that telomere dysfunction in association with p53-dependent premature cell senescence is possibly responsible for the aging of human stem cells.

Telomere shortening and dysfunction are a cause of genomic instability in WS (classic premature aging), resulting from a mutation in a gene coding the RecQ helicase WRN required for telomere maintenance during DNA replication [55].

Previous studies in the role of telomeres in epidermal stem cells observed that deletion of protein binding to telomeric DNA adversely affects skin homeostasis and development at embryonic stages in association with enhanced DNA damage response [56]. In addition, decline in function of ESCs was observed in association with shortened telomeres, which reduced proliferative potential in response to stimuli that may be implicated in the premature skin aging phenotype observed in mice [57]. Conversely, ESCs from aged skin were found to contain elevated levels of chromatin rearrangements and epigenetic changes and indicated that aging might be largely the attribution of structural changes to chromatin, potentially leading to epigenetically induced transcriptional deregulation [58]. Finally, a connection between UVR and telomere attrition has recently been emphasized. A report by Stout and Blasco showed that telomere length is, at least partly, maintained by the same NER pathway that protects DNA from UVR damage. UVR treatment on mouse *XPC* ^−/−^ epidermis resulted in telomere shortening in stem cells [59].

**Figure 1:**
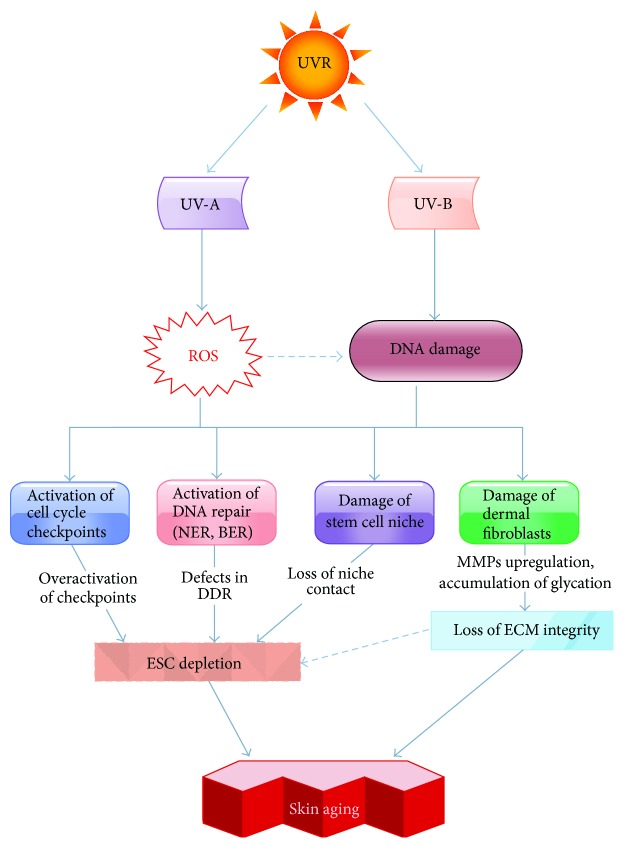
Skin aging induced by UVR-induced DNA damage to ESCs. UVR is the major skin stressor capable of damaging ESC DNA (UV-B) or promoting production of the DNA toxic ROS (UV-A). DNA damaging effects of UVR result in activation of cell cycle arrest (checkpoint activation) and DNA repair proteins, damaged stem cell niche, and dermal fibroblasts. These facilitate ESC depletion and loss of extracellular matrix (ECM) integrity, leading to premature skin aging.

Taken together, these results argue for a strong connection between skin photoaging and DNA damage-induced stem cell exhaustion (Figure 1).

## 4. ESC Exhaustion as a Consequence of Cell Cycle Checkpoint Activation

DNA damage normally triggers activation of checkpoint pathways, simultaneously with the activation of DNA repair mechanisms. Checkpoint proteins such as p53 and ATM are activated upon UVR exposure and they elicit cell cycle arrest signaling to allow proper DNA repair [60]. Deficiencies in the checkpoint pathways result in accumulation of DNA damage lesions, resembling the phenotypes of DNA repair deficiencies. Protein product of ataxia-telangiectasia mutated (ATM) gene, a checkpoint protein that acts as a detecting sensor for DNA damage in a cell, triggers cell cycle arrest/DNA repair. Mutations of the* ATM* gene cause ataxia telangiectasia (AT), an inherited disease associated with premature aging, particularly at the skin. Although not a* bona fide* DNA repair gene, defects in* ATM* lead to accumulation of genomic damage and premature exhaustion of the stem cell pool in various tissues, including the skin [46].

A gene downstream of* ATM*,* p53*, is a transcription factor, which primarily functions as a gatekeeper for DNA mutations [61]. Timely expression of wild-type* p53* is crucial in preventing deregulated or stressed cells from turning into cancer cells [62].* p53* does this by transactivating expressions of gene sets that initiate cell cycle arrest, DNA repair, senescence, or apoptosis. Examples of genes activated by* p53* include* XPE/DDB2* (a gene in the NER pathway) [63],* p21* (a cell cycle inhibitor),* BCL2-associated X protein* (*BAX*), and* TNF* receptor subfamily, member 6 (*FAS*) (both proapoptotic proteins). [64–66]. Upon UV irradiation, p53 is phosphorylated by several kinases, namely,* ATM*,* ATR* (*ATM*-related kinase), and* CHK1/2*. UV-induced phosphorylation at Serine 15 increased* p53* stability and protein level that associated with DNA damage [61]. Inhibition of p53 phosphorylation during UV-induced DNA damage increased sensitivity to UV-induced skin tumor development [67], indicating that p53 activation after DNA damage effectively halts the expansion of cells with damaged DNA, in order to prevent cancer formation.

Interestingly, overactivation or prolonged activation of checkpoint proteins will also result in the diminishing of self-renewal potential of ESCs. Skin of the mouse lacking the negative regulatory protein of* p53*,* MDM2*, elicited increased basal* p53* level and premature aging skin phenotypes, including thinning of the epidermis, reduced wound healing, and progressive hair loss [68].

In agreement with the negative role of prolonged* p53* expression on stem cell potency, it was shown that* p53* restricted the stem cell gene expression program [69]. Suppression of* p53* by viral protein or siRNA facilitated reprograming of iPS [70, 71].* In vivo*,* p53* ^−/−^ mice showed marked stem cell expansion and were prone to teratoma [72]. Lastly, UV-damaged DNA binding proteins 1 (*DDB1*) and 2 (*DDB2*) cooperate with* p53* in DNA damage response. Deletion of* DDB1* in mouse resulted in genomic instability and loss of epidermis, which could be partially rescued by deletion of* p53* [73]. Deletion of* DDB2*, another gene regulated by* p53*, resulted in UV-induced skin cancer [74].

*p63*, a* p53*-related gene, is well accepted as a protein that maintains ESCs. Expression of* p63* selectively distributed in the basal cells of both stratified and glandular epithelia, both of which contained ESCs, suggested its critical role in the regulation of stemness.* p63* and its isoforms are regarded as a marker of ESCs [75]. The transactivating isoform of* p63* (*TAp63*) functions to suppress hyperproliferation of skin stem/progenitor cells that result in exhaustion of the ESC pool or DNA damage.* TAp63*-null mouse skin demonstrates cells with terminal cell cycle arrest (senescence) phenotypes and premature skin aging [76]. Since* p63* was shown to respond to stress signals, similar to* p53*, and is upregulated in prolonged cultured cells, it might be reasonable to expect that* p63* works in DNA damage response and contributes to mediating UV-induced ESC exhaustion [77]. However, there is still no evidence to support this notion.

Checkpoint proteins also triggered DNA repair. In addition to those already mentioned, a mouse model for* BRCA1* deficiency emphasized the significance of unrepaired DNA in stem cell depletion. Deficiency of* BRCA1*, a central protein in single-stranded and double-stranded DNA repair in epidermis is associated with increased DNA damage, genome instability, loss of hair follicle stem cells (HFSCs), and hair follicles. Defects appeared to be the result of activated* p53*, since deletion of* p53* offset the effects of* BRCA1* loss [78].* BRCA1* plays a role in UV-induced DNA damage response. UV-induced DNA damage produced* BRCA1-*positive foci as part of the damage response [79]. Interestingly,* BRCA1* deletion in skin epidermis using* K14-Cre* did not affect stem cells in sebaceous grand and interfollicular epidermis, suggesting that BRCA1 is only indispensable for some populations of stem cells.

## 5. DNA Damage Promotes Photoaging by Depriving Stem Cells of Their Niche

Maintenance and repair of many adult tissues rely on stem cells to produce numerous differentiated cells that replace or repair the tissue [80–82]. Stem cells in the pool spend relatively long period of time in a quiescent state and the pool is to be used throughout the life of the organism. ESCs in the pool will exit from the quiescent stage to divide to produce more stem cells or committed progeny cells, which will then eventually differentiate into various cellular components of skin. Maintenance of the pool relies on extrinsic factors, such as signaling from neighboring cells and surrounding environment (microenvironment) that make up the stem cell niche and cell autonomous regulations [80, 83]. Irradiated quiescent MSCs prematurely differentiate in the niche upon their activation without sufficiently renewing themselves. These data suggest that tissue radiosensitivity may be dependent on the state of somatic stem cells in their local microenvironment [84].

UV-induced DNA damage was also reported to interfere with skin stem cell pool by causing changes in the stem cell niche. ESCs are surrounded and maintained by a microenvironment of various cell types and substances [85]. The homeostasis of stem cells is influenced by cross talk between ESCs and mesenchyme cells and the interaction between stem cells and extracellular matrix (ECM; laminin, type IV collagen, nidogen, and perlecan), the constituents of the basement membrane of skin. Communication between ECM components is mediated by integrins, a family of transmembrane proteins on the ESCs surface. Integrins contact other cells or the ECM. Such connections trigger signal transduction pathways, which result in biological responses, such as cell proliferation or cell motility. The notion that ESCs express abundant levels of integrins indicates that communication with the niche environment is fundamental to ESC stemness (high level of *α*6-integrin is considered one of the hallmarks of ESCs). Fibronectin, vitronectin, collagen, and laminin are all ligands for integrins and have been reported to play an intricate role in ESC homeostasis. It was demonstrated in a study of HFSCs that the mesenchymal niche of the hair follicle provides molecular signals that are sufficient to induce hair growth [86]. Interaction between integrins and ECM is required for stem cell maintenance. Loss of contact from the ECM decreases potency of stem cells [87] and *β*1 integrin-mediated adhesion signaling is essential for epidermal progenitor cell expansion.

DNA damage appears to also activate expression of genes that are not directly implicated in DNA damage response but that play a role in skin stem cell homeostasis. Transcription factor c-Myc is one of the genes found to be activated by UVR.* c-Myc* overexpression was detected approximately 5 hours after UV irradiation of the skin cells [88].* c-Myc* expression was shown to correlate with reduced potency of skin cells. Deregulated* c-Myc* expression was shown to deplete ESCs by making the ESCs overproliferate, move out of their niche, and then differentiate [89, 90].* c-Myc* could also induce ESC differentiation as mediated by* c-Myc* in the c-Myc-Miz1 complex, which supposedly suppresses genes critical for skin cell stemness [91]. In fact, *β*1 integrin was reported to be a gene target for repression by the c-Myc-Miz1 complex in stem cell depleted skin [91]. These results indicated that stem cells favor low level of c-Myc to stay in undifferentiated status, with DNA damage triggering untimely* c-Myc* overexpression, resulting in ESC exhaustion.

In addition, long-term UVR exposure can cause restructuring of the extracellular matrix. Proteins in extracellular matrix components contain UV-absorbing chromophores, which are vulnerable to the UV-induced structural changes [92]. UV-induced changes in ECM are well characterized. UVR exposure not only upregulates the expression of* MMP-1, MMP-2, MMP-3, MMP-7, MMP-9,* and* MMP-12* [93], but also promotes a prooxidative environment in human skin, by which the structure of the skin will be further damaged. Although this restructuring of the extracellular matrix likely disturbs the ESC pool, detailed studies are needed to identify and elucidate the effects.

## 6. UV-Induced Oxidative Stress: A Major Assault on Skin and a Potential Preventive and Therapeutic Target for Antioxidants in Skin Photoaging

UV-A-induced ROS is, to a major degree, responsible for ESCs DNA damage. Normally, reactive oxygen species (ROS) are endogenously produced in the cell from various metabolic processes, including cellular respiration, which takes place in mitochondria and is the primary source of intracellular ROS [94]. However, ROS production can also be a by-product of cell exposure to the environment, including exposure to UVR, ionizing radiation, pollutants, cigarette smoke, alcohol, heavy or transition metals, and certain drugs [95–97]. ROS include not only free radicals, such as superoxide anion radical (O_2_^•−^) and hydroxyl radical (OH^•^), but also nonradicals like singlet oxygen (^1^O_2_) and hydrogen peroxide (H_2_O_2_). ROS play a dual role as both harmful and useful molecules in the cell; as such, an appropriate level of ROS is needed for normal physiological function of mammalian cells and, therefore, is essential for life. Phagocytic ROS act as weapons for the immune system to kill pathogenic microorganisms that cause infection. ROS also take physiological roles in the regulation of several cellular functions and signaling cascades involved in proliferation, differentiation, and survival of various types of cells, such as endothelial cells, fibroblasts, smooth muscle cells, cardiac myocytes, neuronal cells, and skin keratinocytes [98–101].

While ESCs appear capable of adequate responsiveness to changes in their environment, accumulated oxidative damage to biomolecules in the cells, including lipids, proteins, and DNA, caused by repetitive exposure to environmental insults, in particular UVR, may compromise their self-renewal capacity and promote loss of ESC numbers, resulting in premature aging [42, 102]. Although the mechanisms by which ROS act on molecular changes in ESCs in the process of skin aging are not well understood, it has been shown that elevated ROS is responsible for premature aging of the stem cells of the skin. Wang et al. observed that persistent elevation of ROS and oxidative DNA damage are associated with increased senescence and decreased clonogenic capacity of hematopoietic stem cells (HSC) [103].

Thus, it is very important to understand the role of ROS in ESC DNA damage and disturbed ESC homeostasis, in particular, in association with DNA damage. In many cases, the models that contained defect in antioxidation pathways or DNA repair helped to explain the significance of ROS-induced DNA damage during ESC exhaustion process.

## 7. ROS in DNA Damage and Defective DNA Repair Systems Implicated in Loss of Epidermal Stemness

Beyond DNA repair mechanisms, protection of skin cells against UV-induced DNA damage includes enzymatic antioxidants [104]. It has been suggested that stem cell DNA damage is often accompanied by oxidative stress, which is an accountable factor for stem cell senescence and aging [105, 106]. Excessive ROS can result in single-strand break products. Oxidative DNA damage is continuously accumulated throughout the lifespan of a living cell. During physiological aging, DNA is constantly exposed to intracellular ROS, as by-products of metabolic process. ROS can introduce DNA base or sugar damage resulting in formation of approximately 5000 single-strand breaks in each cell in the human body daily [107, 108].

ROS similarly damage both nuclear and mitochondrial DNA (mtDNA). It was recently reported that aging of the skin was observed to be associated with mtDNA damage and mutations. mtDNA mutations were suggested as being a sensitive biomarker of UVR exposure and oxidative stress in human skin [109]. Additionally, our study previously demonstrated that induction of oxidative DNA damage and defects in DNA repair hOGG1 that could be responsible for photocarcinogenesis were associated with patients with BCC [110]. Another report showed that overproduction of ROS (generated during ionizing radiation) was involved with MSC depletion in graying hair, an obvious sign of aging. Detailed study showed that ROS-induced DNA damage led to MSC attrition and discontinued renewal of MSCs in mice by enhancing MSC differentiation into mature melanocytes. The same study proposed that ATM was the sensor of ROS-induced DNA damage and acted as a checkpoint for the stemness. Deficiency of ATM from MSCs stimulated MSC differentiation [111]. In another study, ROS-induced depletion of HSC was shown to be mediated by p38 MAPK phosphorylation in the *ATM* ^−/−^ cells [112].

Integrity of chromosomes is determined by the length of the telomere. Cellular aging is associated with shortened telomere. Adequate telomerase (TERT) activity is required to maintain telomere length. DNA damage in association with telomere dysfunction and shortening precipitates premature aging and shortened lifespan, leading to depletion of almost all tissue stem cell reserves and dysfunction of stem cells, including self-renewal and repair capacity of ESCs and melanocytes of the follicle during the hair growth process [113, 114]. Interestingly, high telomerase expression, which is associated with long telomeres, is commonly found in adult stem/progenitor cell compartments, including the stem cells in hair follicles, whereas low telomerase expression has been detected in human fibroblasts and epithelial cells [115, 116].

ROS may also activate the p53-p21 axis, Akt-MAPK, ATM pathways [117], and forkhead homeobox type O (FOXO) transcription factors in various types of stem cells by influencing telomere length [118]. Moreover, the reduced proliferative potency of stem cells was induced by ROS-activating p53 stabilization [119]. Skin aging associated with ROS accumulation, thus, was found to be rescued in the skin of late-generation p53 knockout mice [120]. In these mice, there were improved wound healing, hair growth, and skin renewal to levels comparable to those of wild-type mice, as well as increased ESC numbers and improved mobilization capacity [120].

Effects of ROS on supportive cell types have also been studied. Exposure of human skin fibroblasts, an important cellular component of skin microenvironment, to UVR has been shown to result in oxidative stress and enhanced expression of cell cycle inhibitors* p16INK4A* and* p53/p21WAF1/CIP1*. Upregulations were predictably followed by premature senescence of fibroblasts [121, 122], which unavoidably disturbed stem cell-niche cross talk [123]. High level of ROS was also reported to induce skin fibroblasts to secrete high levels of matrix metalloproteinases (MMPs), which caused extensive ECM remodeling in dermal layers [124–126].

In addition to the direct DNA damage caused by UVR, ROS, a by-product of UV-A exposure, also plays prominent role as a DNA damaging agent that damages DNA in an equally extensive manner. However, due to the transient nature of ROS, it is possible to ameliorate its effect and protect the skin from premature aging. Pharmacologically, ROS has been considered as a potential target for photoaging prevention and therapies.

## 8. Preventive and Therapeutic Implications of Antioxidants in Photoaging through Preservation of Stemness

Regarding the importance of stem cells, several protective mechanisms have been detected in different stem cell types for protecting against ROS and UV-induced DNA damage [127, 128].

At present, strategies to potentiate DNA repair pathway or to restore functions of defective NER proteins are under investigation. Gene therapies for the XP might be possible with the emergence of gene editing technologies [129, 130].

Currently, prevention and treatment strategies for photoaged skin mostly center on strengthening antioxidant defense for the cells. Mammalian tissues are equipped with an array of nonenzymatic and enzymatic antioxidant systems that work in a complex network to maintain the optimal level of ROS in the cells or a redox balance by several mechanisms. These mechanisms work to neutralize oxidants, inhibit oxidative damage, and regulate transcription factors involved in redox and scavenging reactions [131]. Proliferation and differentiation of human cells underlie a delicate control of several signaling pathways [132]. The balance of redox homeostasis and regulation of ROS-mediated signaling are part of the central regulation involved in self-renewal, proliferation, and differentiation of normal stem and progenitor cells in human tissues, including the skin [133].

Since skin cells can be exposed to environmental sources of ROS, particularly UVR, endogenous antioxidants function as a crucial defense system to preserve redox balance required for regulation of epidermal homeostasis and prevent ROS-induced skin damage [134]. Important endogenous antioxidants include enzymatic antioxidants, such as superoxide dismutase (SOD), catalase (CAT), glutathione peroxidase (GPx), glutathione reductase, and thioredoxin reductase, and nonenzymatic antioxidants or low molecular weight antioxidants, such as ascorbate or vitamin C, glutathione (GSH), and *α*-tocopherol or vitamin E [135]. SOD is an important antioxidant enzyme that converts O_2_^•−^ into H_2_O_2_, which is finally degraded into water by CAT and GPx.

However, when generated in excess, ROS promote oxidative stress that can damage cellular structures and biomolecules, including lipids, proteins, and DNA, as well as interfering with normal signaling cascades. It has been proposed that an imbalance between ROS production and antioxidant defense would occur as a consequence of overwhelming ROS production and the substantial reduction in antioxidant levels that occurs with advanced aging [136, 137]. On the other hand, the effect of excessive ROS on total lifespan was implicated in a study, which showed that whole body deletion of the SOD1 gene caused a reduced lifespan in mice that died by “aging dead,” suggesting that the physiological level of SOD1 is required to prevent premature aging [138]. Mitochondrial ROS has also been suggested to be a driving force of accelerated aging caused by ROS damage in adult stem cells [139]. Lastly, oxidative stress may also have an impact on the aging process by causing biochemical and physiological changes that accelerate age-related diseases [140].

ROS may be the leading cause of physiological change in skin stem cells. It has been proposed that the maintenance of self-renewal and differentiation capacity of ESCs into old age and their resistance to cellular aging may be attributed to their ability to control levels of intracellular ROS by antioxidant defenses. Consistent with this theory, prevention of oxidative stress, thus, is the key to prevent stem cell exhaustion in the skin [141]. In nature, SOD may be an important antioxidant enzyme that is responsible for the tolerance of ESCs to ROS-mediated aging process, because the SOD gene was observed to be highly expressed in ESCs isolated from both young adult mice and old adult mice and its expression remained unchanged in the ESCs from old mice [20].

Stem cells undergo a self-renewal process that produces low levels of intracellular ROS as by-products. Although these ROS are endogenous and low in amount, they are still harmful to stem cells. In order to maintain redox homeostasis, antioxidant defense systems are transcriptionally upregulated in response to ROS formation. As such, attempts have been made to explore the role of transcription factors that can serve as redox regulators in the self-renewal of stem cells, in particular, FOXO family and nuclear factor erythroid-2-related factor 2 (Nrf2) [142]. Activated FOXOs translocate into the nucleus and mediate transcription of target antioxidant genes, including CAT and SOD [143]. Disruption of FOXO proteins, which are key downstream components of the PI(3)K pathway, was associated with depletion of tissue stem cells without external stimulation [144, 145].

Nrf2 has been widely accepted as the key regulator of redox homeostatic genes involved in human skin adaption to oxidative stress, which can stimulate its translocation into the nucleus to bind to the antioxidant response element (ARE), a* cis*-acting enhancer sequence in the region of several genes encoding antioxidant/detoxification enzymes, including glutamate cysteine ligase (*GCL*), heme oxygenase-1 (*HO-1*), glutathione transferase (*GST*), and NAD(P)H quinine oxidoreductase-1 (*NQO1*) [146–148]. Depletion of Nrf2 can compromise the abilities of various cells (such as mouse embryonic and human primary skin fibroblasts and keratinocyte HaCaT cells) to protect against photooxidative damage [149–152]. Moreover, Nrf2, which may be a target of keratinocyte growth factor, was observed to play a role in cutaneous wound repair in mice in association with an increase in mRNA expressions of* collagen* and* VEGF* (vascular endothelial growth factor), a main regulator of angiogenesis [153]. UV-B-induced apoptosis of basal keratinocytes in murine epidermis was blocked by suprabasal keratinocytes capable of controlling their survival through Nrf2-regulated antioxidant defenses including GSH [154].

**Figure 2:**
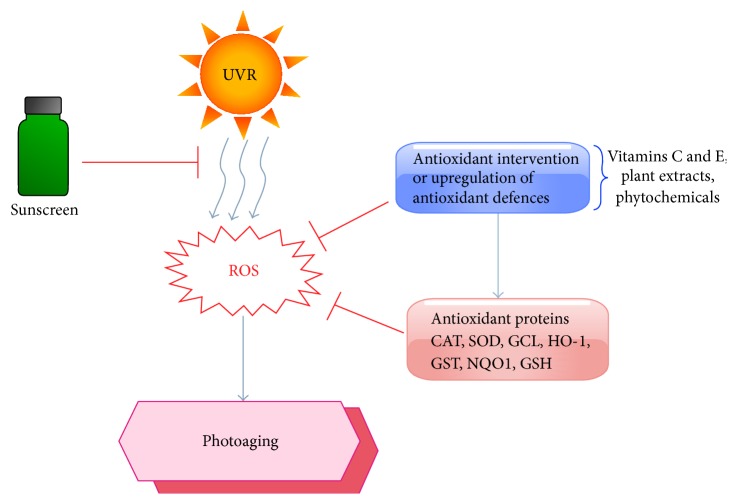
Anti-ROS as a potential anti-skin aging intervention. Possible interventions that delay ESC DNA damage, by preventing UVR exposure using sunscreen, anti-ROS, or upregulation of antioxidant proteins.

While melanin and enzymatic antioxidants serve as endogenous defense of the skin against UVR, accumulative exposure to UVR can overwhelm the ROS defense mechanism. Exogenous and dietary antioxidants such as vitamin C (ascorbic acid), vitamin E (*α*-tocopherol), beta-carotenes, and different phytochemicals, particularly polyphenols derived from plants-based food and beverages, as well as herbal products that play a crucial role in maintaining redox homeostasis and antioxidant intervention are thus proposed as being useful in delaying skin photoaging and helping to prevent skin cancer [104, 155] (Figure 2).

As UV-mediated oxidative stress can adversely affect ESCs, enhancing capacity of cellular antioxidant defenses to counteract oxidative stress would represent a promising strategy for inhibition of photoaged skin. A number of studies using skin cell culture reconstructed skin and animal models have reported protective roles of natural and synthetic antioxidants, as well as medicinal plants against UV-mediated skin photoaging through modulation of antioxidant defense system. For example, compounds capable of activating Nrf2 were able to prevent UV-B-dependent cell death in primary keratinocytes [156] and tanshinones derived from medicinal plant* S. miltiorrhiza*, which acted as Nrf2 inducer, suppressed UV-induced damage of human skin cells, and reconstructed human skin through upregulation of* Nrf2* and its target antioxidants including* GCL* and* NQO1* [157]. Purified* T. parthenium* extracts inhibited UV-mediated DNA damage through activation of Nrf2-ARE signaling in primary human keratinocytes [158]. Eckstolonol isolated from* E. cava* was shown to protect keratinocyte HaCaT cells against UV-B-mediated photooxidative stress, cytotoxicity, and DNA damage possibly through induction of CAT and SOD activities [159]. 18*β*-glycyrrhetinic acid, the major active ingredient in licorice root, inhibited UV-induced skin photoaging in a mouse model by induction in skin collagen content and downregulation of MMP-1 and MMP-3 associated with increased activities of SOD and GPx [160]. Furthermore, the mechanisms by which oral administration of* Polypodium leucotomos* delayed skin tumor development in hairless mice might involve improvement in antioxidant defense status [161].* K. parviflora* (black ginger) extracts orally administrated to hairless mice exerted antiphotoaging effects on UV-B-irradiated hairless mice by suppression of MMPs and upregulation of collagen genes in association with induction of CAT expression [162]. We have also reported the protective roles of antioxidant phytochemicals, such as caffeic acid, ferulic acid, and gallic acid, which are ubiquitously present in plants-based food and beverages, against UV-A-induced melanogenesis in different melanoma cell lines and MMP-1 upregulation in HaCaT cells in association with promotion of Nrf2-related antioxidant defenses, including CAT and GSH antioxidant system (GST and GPx) at the cellular and molecular levels [163–166]. Nevertheless, few studies have addressed whether targeting ROS and antioxidant mechanisms could be a promising strategy for the delay of premature skin aging by promoting the capacity of skin stem cells for skin repair and rejuvenation.

Choi et al. suggested that redox status is essential for stemness in skin equivalents and demonstrated that vitamin C, plant extracts (*G. lucidum* and* R. sachalinensis*), and a novel tripeptide “ACQ: alanine-cysteine-glutamine” having high antioxidant activities promoted stemness and the proliferative capacity of epidermal basal cells by improving microenvironment through regulation of integrins, the main receptors for ECM [167, 168]. In addition to various antioxidant compounds reported as having beneficial effects on stemness of the skin, the use of adult stem cells and their niches to contribute to regeneration and repair of damaged tissues has been proposed as a promising strategy in cell-based therapy in tissue injury. Mesenchymal stem cells within the stromal-vascular fraction of subcutaneous adipose tissue successfully employed to substitute dermal fibroblasts for skin construction were demonstrated to improve UV-B-induced wrinkles in hairless mice and exert antiapoptotic effects on dermal fibroblasts through antioxidant mechanisms, including upregulation of various antioxidant proteins [3, 169].

Notably, pharmacologic agents with antioxidant actions and tissue engineering approaches have been proposed to promote stemness of the skin, although their efficacy and safety for clinical application in prevention and treatment of skin photoaging warrant further investigation.
